# Resection of a Rare Metacarpal Distal Condyle Osteoid Osteoma

**DOI:** 10.1155/2019/4542862

**Published:** 2019-05-26

**Authors:** Bachar El Fatayri, Az-Eddine Djebara, Alex Fourdrain, Yassine Bulaid, Mario Sanguina

**Affiliations:** ^1^Orthopedic Surgery Department, CHU Amiens-Picardie, 80480 Salouël, France; ^2^Orthopedic Surgery Department, CHG Clermont de l'Oise, 60607 Clermont de l'Oise, France

## Abstract

**Introduction:**

Osteoid osteoma is a benign bone-forming tumor with young male predilection. It occurs predominantly in the long bones. In the hand, osteoid osteoma is more commonly located in the phalanges and carpal bones. The metacarpals are the least common site for osteoid osteoma. Pain is the most common symptom. It usually increases at night and responds to nonsteroidal anti-inflammatory drugs.

**Case Presentation:**

The authors report the excision of an osteoid osteoma lying at the distal condyle of a metacarpal bone of the left hand. The clinical and radiological findings are exposed as well as the surgical management of the lesion. Pain and swelling disappeared after surgery, and there was no evidence of recurrence at follow-up.

**Discussion:**

They discuss this rare location and further radiological examination that was used leading to the diagnosis. The imputation of the traumatic factor has been discussed, along with the different therapeutic possibilities and the advantages of a total excision while preserving the integrity of the adjacent ligament and joint space.

**Conclusion:**

The surgical alternative appears to be a satisfying treatment for osteoid osteoma in this particular superficial location. Both the exact pathogenesis and the contribution of the traumatic factor remain unclear.

## 1. Introduction

Osteoid osteoma (OO) is a benign bone tumor. In 1935, Jaffe studied it and realized it has no infectious origins [1]. OO is relatively frequent; it represents 2 to 3% of the primitive bone tumors and 12% of all benign bone neoplasia [2, 3]. Male patients are more often affected than females by a ratio of 2 : 1, and the tumor principally occurs between 7 and 25 years [3, 4].

The tumor appears as an oval lytic lesion called “nidus,” surrounded by fusiform cortical bone thickening. The nidus ranging between 5 and 20 mm is inconstantly visualized due to significant sclerosis [5].

OO can emerge in various locations, principally in the long bones of the lower extremity (essentially the femur and tibia). It is rarely localized in the hand, where it occurs most frequently in the phalanx. The metacarpal bone is the least common site for OO [2, 3, 6]. The predominant location is cortical, where the “nidus” resides in a fusiform cortical thickening, usually in the diaphysis or metaphysis. Cancellous and subperiosteal tumors usually frequently arise in juxta-articular locations [2, 3].

Chronic unexplained striking pains that worsen at night and promptly relieved by nonsteroidal anti-inflammatory drugs (NSAIDs) dominate the classic symptomatology [7]. The pain is located nearby the tumor. Swelling, an inconstant sign, occurs especially when the concerned bone is beneath the skin [5, 8]. This edema is the consequence of the high vascular nature of the tumor. Other signs have been described, depending on the tumor's topography, like swelling, stiffness, and effusion in the joint. There are other painless cases of OO, particularly described in the finger and toe phalanges [9, 10].

Nowadays, the pathogenesis of this lesion stays ambiguous. Some authors think that it is an inflammatory regenerating process, while others suggest it to be a benign neoplasm process [5, 11]. Nevertheless, its improbable malignancy is agreed upon. In accordance with its scarcity in metacarpal bones, we report on clinical and radiological findings of an unusual topography of OO within a metacarpal distal epiphysis, and then we discuss its surgical management and results.

## 2. Case Presentation

Our 32-year-old right handed patient was first examined in a consultation and ambulatory care unit because of chronic pains adjoining the head of the second metacarpal bone of the left hand, evolving over the past 6 months. The patient is recorded to have had a left hand trauma 7 years ago. The pain is characteristic, increasing at night and partially relieved by aspirin. Neither sensory disorder nor functional disability is found. There was a medial posterior swelling of the second metacarpophalangeal joint. Palpation and motion awaken the pain.

**Figure 1:**
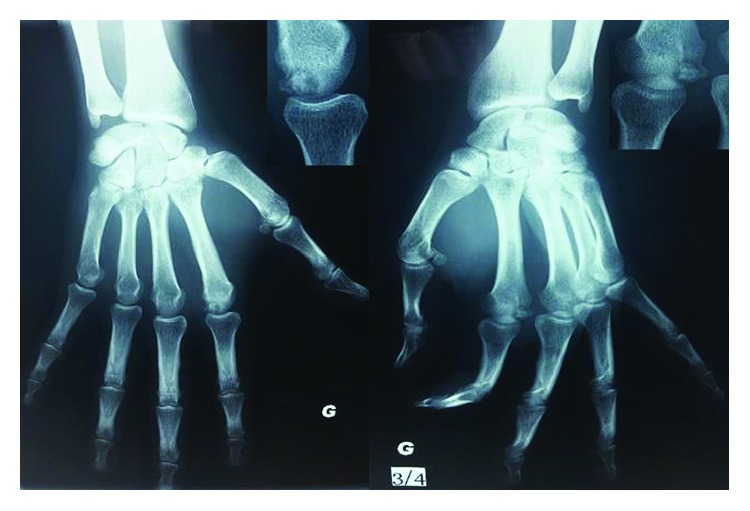
Left hand radiographs with illustrative zoom.

The prescribed X-rays have shown a distal epiphysis subchondral lesion of the second metacarpal bone.  Figure 1 shows the left hand radiographs in addition of an illustrative zoom highlighting a small, nodular, irregular, and radiolucent lesion, on the medial distal condyle, with significant sclerosis.

**Figure 2:**
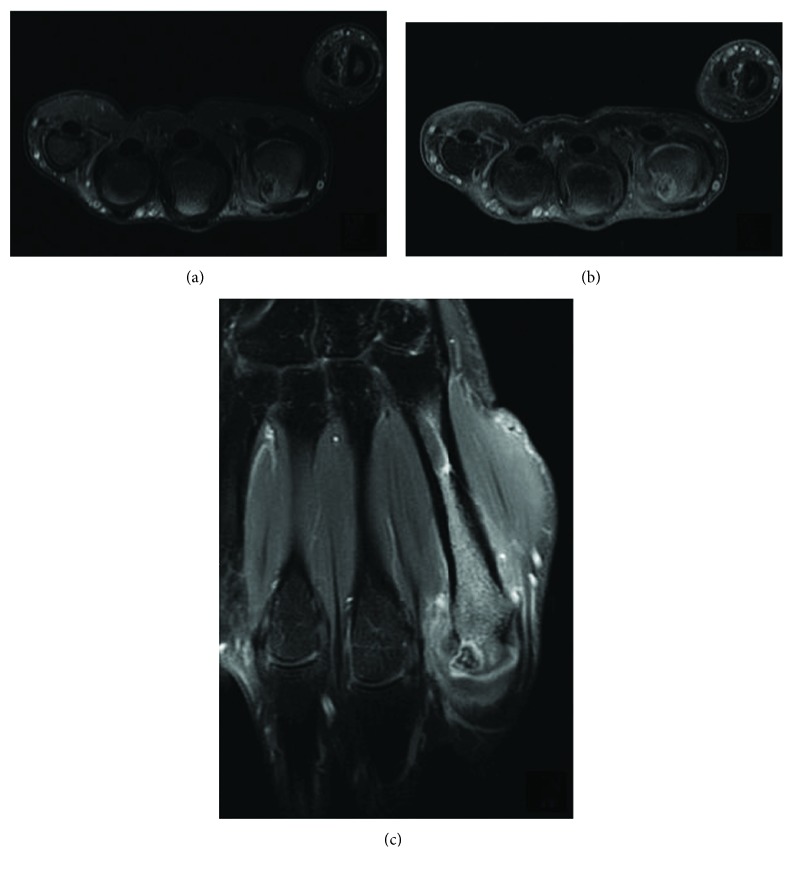
Nuclear magnetic resonance imaging (NMRI). (a) Axial fat-suppressed T2-weighted NMRI. (b, c) Respectively, axial and coronal, gadolinium-enhanced T1-weighted NMRI.

An additional morphological examination by nuclear magnetic resonance imaging (NMRI) concluded to a high compatibility with the diagnosis of OO.  Figure 2(a) (axial fat-suppressed T2-weighted NMRI) shows a nodular low-signal intensity lesion, surrounded by a high-signal intensity reactive edema. Figures 2(b) and 2(c) (respectively, axial and coronal, gadolinium-enhanced T1-weighted NMRI) bring to light a strong enhancement of the tumor and the medullary bone, in addition of the adjacent soft tissue.

A surgical gesture consisting of a simple *en bloc* excision of the tumor without bone restauration has been proposed. Surgery took place under a brachial plexus block and was performed via a medial dorsal approach. A complete excision of the nidus was undertaken using an oscillating saw associated with the removal of the sclerotic bone inside the lesion with a curette.

**Figure 3:**
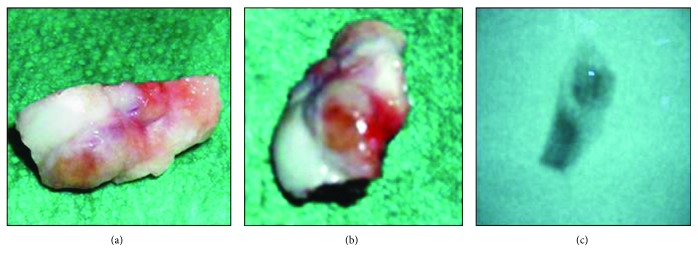
Operative specimen with its intraoperative radiograph.

The excision respects the collateral medial ligament of the second metacarpophalangeal joint. A small dorsal portion of the cartilage, judged insignificant, had to be removed along with the tumor. The operative specimen was controlled with a brilliancy amplifier as a complement of the quality of a total tumorectomy. Then, the specimen was sent for histological examination.  Figure 3 reveals the operative specimen with its intraoperative radiograph.

**Figure 4:**
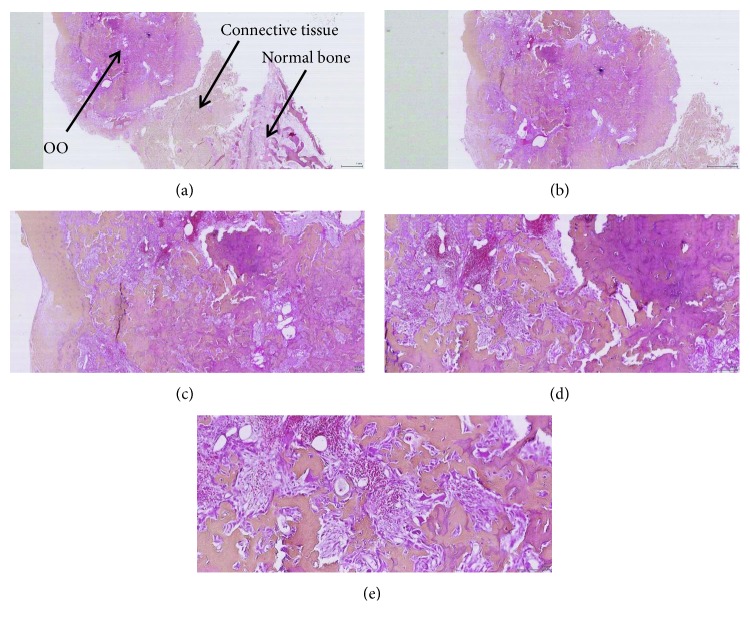
Histological examination (magnification factor: (a) ×10, (b) ×20, (c) ×40, (d) ×100, and (e) ×200). (a) OO separated from the normal bone by connective tissue. (e) Osteoid and woven bone with interconnected trabeculae and a background and rim of highly vascularized connective tissue.

Histological analysis confirmed the diagnostic hypothesis. It shows, as referred to in Figure 4, a condensation zone constituted of irregular trabecula of the bone, surrounded in periphery by a high vascular connective tissue, equally containing disordered trabecula of the bone. The histologic diagnosis is in favor of an OO with total resection.

After surgery, the left hand was immobilized with a splint for 3 weeks, then with a second-third finger syndactyly for another 3 weeks, associated with simple painkillers.

**Figure 5:**
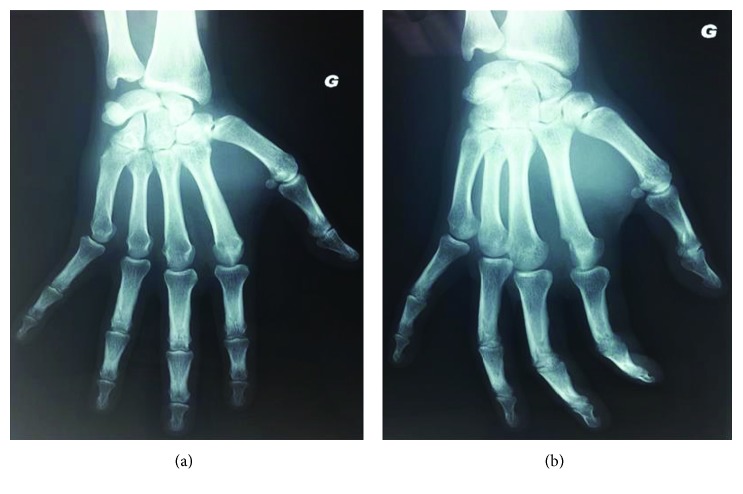
Postoperative radiographs.

At six-week follow-up, the pain has disappeared as well as the swelling. The X-rays have shown total excision and a respected joint space.  Figure 5 points out postoperative radiographs. At 6- and 12-month follow-up appointments, the patient was pain free and had normal motion. He stopped consuming NSAIDs and resumed manual labor and daily activities. There were no signs of recurrence and X-rays remained unchanged.

## 3. Discussion

The case being discussed had the necessary clinical criteria to suspect the OO diagnosis. The different elements leading to this diagnosis are constantly reported in the literature: chronic unexplained pain in 80% of the cases, with extreme striking pain nocturnally, and inconstantly, localized swelling, synovitis, and joint stiffness.

Pain is the predominant sign. Mungo et al. [12] relate the fact principally to high levels of prostaglandins in the nidus (100 to 1000 times superior than the average in a normal bone). These inflammatory mediators privilege the appearance of edema by acting on vascular permeability, therefore understanding the efficiency of NSAIDs that inhibit the production of prostaglandins [13].

The documentation of this pathology was done through the use of standard radiographies and NMRI, leading to an excellent visualization of the lesion, its limits, and the edema around it. The choice of NMRI as an additional morphological test was based on its perfect characterization of the lesion, reinforced with the injection of a contrast product, as described by Kawaguchi et al. [14].

There was a delay before reaching a hypothesis of OO due to the atypical location of the lesion and the multiple differential diagnoses at this site.

As illustrated above, OO occurs predominantly in the long bones of the lower body [2, 3]. In the hand, it represents only 8% of the described cases. Many articles of the literature report it in the hands [15–20]. In reference to these works, OO arises mainly in phalanges but is also commonly found in carpal bones. The metacarpals are the least common sites for OO, thus the interest in this special case. A case with an epiphyseal subchondral topography OO of the metacarpal bone has never particularly been reported.

In addition to this rare occurrence, the patient had a history of a left hand trauma 7 years before he became symptomatic. However, no consensual agreement exists linking the benign tumor to a previous injury. Nevertheless, there are numerous works that underline the existence of a trauma before OO diagnosis, with variable time intervals between the trauma and the pain symptomatology debut [21–24]. The high incidence of traumas, especially of the hand, comparative to the much lower incidence of OO, suggests the weakness of this correlation. This finding is strengthened by the variable character of the time interval between the initial trauma injury and the benign tumor diagnosis.

Multiple treatment options have been described. Nonoperative treatment can be considered in cases with good response to aspirin or other NSAIDs [25, 26]. Nonetheless, it is preferable to consider other therapeutic options in severe cases, regarding the potential side effects of protracted NSAIDs or when these drugs are contraindicated. Curettage is the traditional approach for these tumors. Other available procedures are *en bloc* resection and percutaneous techniques using CT guidance. The latter is preferentially used in deep bones, to reduce surgical morbidity and time for recovery. These techniques include trephine excision, cryoablation, radiofrequency ablation, and laser thermocoagulation [27–31].

The option chosen for this case was a surgical treatment, consisting of a total excision of the tumor, while preserving the lateral ulnar metacarpophalangeal ligament and without bone restauration. This choice seemed logical knowing that metacarpals are beneath the skin with simple surgical access. This choice permitted a clinical and functional recovery ad integrum, authorizing the patient to return to his daily activities without limitations.

## 4. Conclusion

The distal epiphyseal topography in metacarpals is a rare site for OO. A typical symptomatic presentation, as well as a sensibility to NSAIDs, should raise the specter of this benign tumor with the most frequent differential diagnosis in hand pathologies. Among the therapeutic arsenal at our disposal, the surgical alternative respecting the adjacent ligaments and the joint space appears to be a satisfying treatment for OO in this particular superficial location. Minimally invasive techniques with CT guidance have shown satisfactory outcomes in deep locations. Until this day, the pathogenesis of OO remains to be discussed, including the contribution of the traumatic factor in its development.
